# Ketorolac salt is a newly discovered DDX3 inhibitor to treat oral cancer

**DOI:** 10.1038/srep09982

**Published:** 2015-04-28

**Authors:** Sabindra K. Samal, Samapika Routray, Ganesh Kumar Veeramachaneni, Rupesh Dash, Mahendran Botlagunta

**Affiliations:** 1 Institute of Life Sciences, Bhubaneswar-751023, Odisha, India; 2 Manipal University, Manipal-576104, Karnataka, India; 3 Department of Oral Pathology & Microbiology, Institute of Dental Sciences, ‘Siksha O Anusandhan’ University, Bhubaneswar-751003, Odisha, India; 4 Biomedical Research Laboratory, Department of Biotechnology, KLEF University (Koneru Lakshmaiah Education Foundation) Vaddeswaram, Guntur, Andhra Pradesh, India; 5 Sweety Biologicals India Private Limited, VengalRao Nagar, Kavali-524201, Andhra Pradesh, India

## Abstract

DDX3 belongs to DEAD box RNA helicase family and is involved in the progression of several types of cancer. In this work, we employed a High Throughput Virtual screening approach to identify bioactive compounds against DDX3 from ZINC natural database. Ketorolac salt was selected based on its binding free energy less than or equals to −5 Kcal/mol with reference to existing synthetic DDX3 inhibitors and strong hydrogen bond interactions as similar to crystallized DDX3 protein (2I4I). The anti-cancer activity of Ketorolac salt against DDX3 was tested using oral squamous cell carcinoma (OSCC) cell lines. This compound significantly down regulated the expression of DDX3 in human OSCC line (H357) and the half maximal growth inhibitory concentration (IC_50_) of Ketorolac salt in H357 cell line is 2.6 µM. Ketorolac salt also inhibited the ATP hydrolysis by directly interacting with DDX3. More importantly, we observed decreased number of neoplastic tongue lesions and reduced lesion severity in Ketorolac salt treated groups in a carcinogen induced tongue tumor mouse model. Taken together, our result demonstrates that Ketorolac salt is a newly discovered bioactive compound against DDX3 and this compound can be used as an ideal drug candidate to treat DDX3 associated oral cancer.

RNA helicases are distinct family members found in all eukaryotes and in majority of prokaryotes^1^,^2^. These members are distinguished from others based on conserved amino-acid sequence Asp-Glu-Ala-Asp/His (DEAD/H)^3^,^4^. These proteins have shown to be associated with numerous aspects of RNA metabolism and translation^5^,^6^, Among several DEAD box RNA helicases, DDX3 (also known as *DBX* and *CAP-Rf*) is located on X-chromosome at Xp11.3-p11.23^7^. Human DDX3 encodes a transcript (5.3 kb) that translates into a polypeptide of 662 amino acids^1^,^8^. DDX3 was crystallized with the help of Adenosine Mononucleotide (AMP). Crystallized DDX3 has two distinguishable domains comprised of N-terminal DEAD box domain 1 (211-403 residues) and C-terminal helicase domain 2 (411-575 residues). Both domains displayed RecA-like folds comprising a central β-sheet flanked by α-helices connected by a non-canonical linker of 11 amino acids^9^. Expression of DDX3 was detected in several tissues^8^,^10^, indicating the universal role of DDX3 in cellular homeostasis. We have previously reported that over-expression of DDX3 in immortalized non-turmorigenic MCF10A cells promoted neoplastic transformation as indicated by down regulation of a cell adhesion molecule E-cadherin^11^. Down-regulation of E-cadherin is a common feature of variety of metastatic epithelial tumors, including those of the lung, breast and prostate cancer^11^,^12^,^13^,^14^. Hypoxic regions of solid tumors were considered to be the primary sites for the generation of the metastatic phenotype^15^,^16^,^17^,^18^. We have also reported that hypoxia inducible factor (HIF-1) induce the expression of DDX3 by binding directly or indirectly to the hypoxia-response element (HRE) in the DDX3 proximal promoter^19^. On the other hand, a significant down regulation of DDX3 expression is found in hepatocellular carcinoma (HCCs) from the hepatitis B virus (HBV) positive patients^20^, In the hepatocellular carcinoma model, DDX3 found to act as a tumor suppressor by activating the expression of cyclin dependent kinase inhibitor p21^cip1^^21^. Besides cancer, expression of DDX3 also found in HIV-1 infected cells^22^,^23^. Overall, it suggests that DDX3 is a multifunctional protein and it plays critical role in cell signaling pathways.

In this paper we validated the biological activity of Ketorolac salt ((±)-5-benzoyl-2,3-dihydro-1H-pyrrolizine-1-carboxylic acid, tris (hydroxymethyl) amino methane salt) against oral cancer. Our results showed that Ketorolac salt effectively inhibited the growth of H357, a human OSCC cell line. Our *in-silico* data suggests that Ketorolac salt forms stable hydrogen bond interactions with Gly 227, Gly 229, Thr 231 and Ser 228 of DDX3 receptor. We further found that Ketorolac salt down regulated DDX3 expression and up regulated the expression levels of E-cadherin protein in OSCC cell line. Along the lines, we also observed that Ketorolac salt reduced tongue lesions in *in-vivo* mice models of oral cancer. Taken together, our result demonstrates that Ketorolac salt inhibits DDX3 expression and this compound can be used as an ideal drug candidate to treat DDX3 associated oral cancer.

## Results

### Virtual screening for the identification of natural small molecule Inhibitors against DDX3

**Figure 1:**
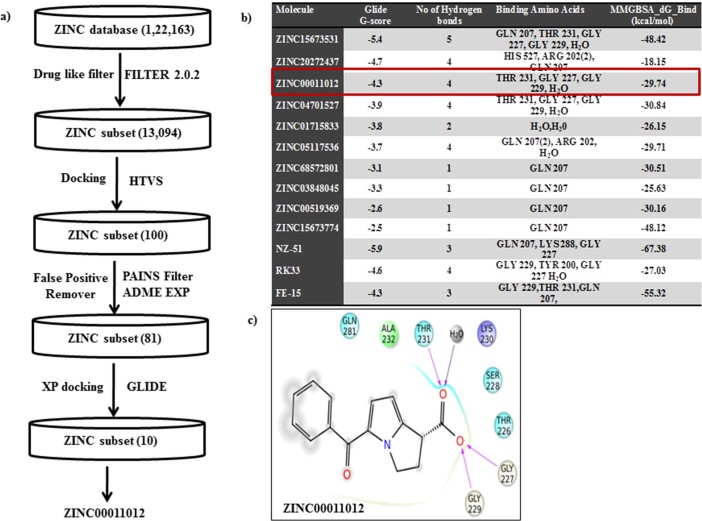
Virtual screening for the identification of natural Inhibitors against DDX3 a) A flow diagram depicting a step wise procedure employed for the virtual screening of bioactive compounds against DDX3 and b) the calculated energy values to the top 10 hit compounds was depicted in tabular form c) The ligand interaction is depicted in the binding pocket of the target protein (2I4I) along with hydrogen and non-hydrogen bond interactions.

To identify the bioactive compounds against DDX3, a set of 1, 22,163 commercially available bioactive molecules were collected from a ZINC database (https://zinc.docking.org/browse/catalogs/natural-products), and they were passed through FILTER 2.0.2 to remove undesirable non-lead like compounds using the default filter lead parameter file (OpenEye Scientific Software v. 2.0.2). Subsequently the total compound entries were reduced to 13,094. These ligands were further subjected to standard-precision (SP) rigid docking protocol in the Schrödinger suite for high throughput virtual screening (HTVS) to identify the compounds that fit into a receptor cavity site of DDX3 (wild-type, PDB code: 2I4I). A total of 100 ligands were selected based on the glide score and they were passed through Pan-assay interference compounds (PAINS) substructure filter. This filter passed 81 compounds and they also showed good absorption, distribution, metabolism and excretion (ADME/Tox) drug properties (Supplementary Table 1). The ADME passed ligands were docked using extra precision *(*XP*)* modes of Glide in Schrödinger 9.6 Suite. Ten compounds with binding free energy less than or equal to −5 Kcal/mol with reference to synthetic drugs FE-15, RK33 and NZ-51 and strong hydrogen bond interactions as similar to crystallized DDX3 protein (2I4I) were further considered for anti-cancer activity. The virtual screening, selection process of bioactive compounds against DDX3 and the calculated energy values to the top 10 hit compounds are depicted in (Figure 1a and 1b). The binding energy between DDX3 and Ketorolac salt (ZINC00011012) is −29.74 kcal/mol, which is nearer to the binding energy of DDX3 and RK33 complex (−27.03 Kcal/mol). This small difference between binding free energy values and G-Scores values suggest that Ketorolac salt and RK33 bound to DDX3 with relatively equal binding affinity. In our docking analysis Ketorolac salt was found buried deep within a narrow pocket formed by the inner lobe cleft as reported to X-ray crystallographic structure (Figure 1c)^9^. Ketorolac salt showed a lowest binding energy of −4.3 K.cal/mol and three direct hydrogen bonds with Glycine 227, Glycine 229 and Threonine 231 aminoacid residues. In addition, one more hydrogen bond interaction was detected in the presence of water molecules between the 18^th^position of Ketorolac salt and OH-609 of water molecule.

### Analysis of DDX3 expression in OSCC cell lines

**Figure 2:**
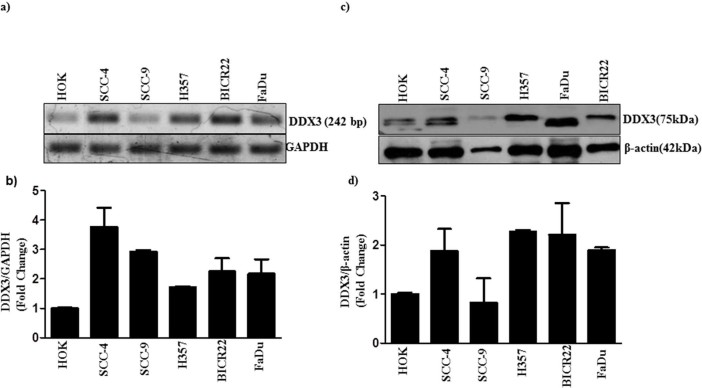
DDX3 expression in human OSCC: a) Total RNA was isolated from indicated cells and RT-PCR was performed to analyze the expression of DDX3 as described in materials and methods. b) The Bar graph indicates the ratio of DDX3 to GAPDH band intensity. SD (n = 3). c) Cell lysates were collected from the indicated cells, and expression of DDX3 was determined by Western blotting. d) The Bar graph indicates the ratio of DDX3 to β-actin band intensity. SD (n = 3).

To determine whether the expression of DDX3 could correlate with oral cancer progression, we analyzed the mRNA levels of DDX3 expression using a panel of Oral Squamous Cell Carcinoma (OSCC) cell lines with varying degree of invasiveness and the normal counterpart human oral keratinocyte (HOK). FaDu is a human pharynx SCC cell line. Figure 2a shows that the expression of mRNA is higher in SCC as compared to HOK cells. Magnitude of DDX3 mRNA expression among SCC cell lines is quantified by measuring the signal intensity of the DDX3 with reference to the internal standard GAPDH using NIH ImageJ software. Figure 2b, shows the magnitude of DDX3 expression across the oral cancer cell lines is as follows SCC-4>SCC-9>BICR22>FaDu> H357>HOK. Next, we analyzed the protein levels of DDX3 on the same series of cell lines using DDX3 specific antibodies. Figure 2c shows that the H357 and FaDu cell lines with highest amount of protein in respect to other cancer cell lines and compared to HOK. In addition, we have noticed a variation in the Molecular weight of DDX3 among cancer cell lines. It is interesting to note that some cell lines (HOK and SCC4) show high and low molecular weight signals and others show a single signal with high molecular weight. To address whether mRNA expression correlates with protein expression, we quantified the magnitude of DDX3 protein expression. As shown in Figure 2d the protein expression as follows, H357>FaDu>BICR22>SCC-4>SCC-9>HOK. It suggests that the mRNA transcript levels do not correlate with protein levels.

### In-vitro validation of Ketorolac salt in oral cancer cell line

**Figure 3:**
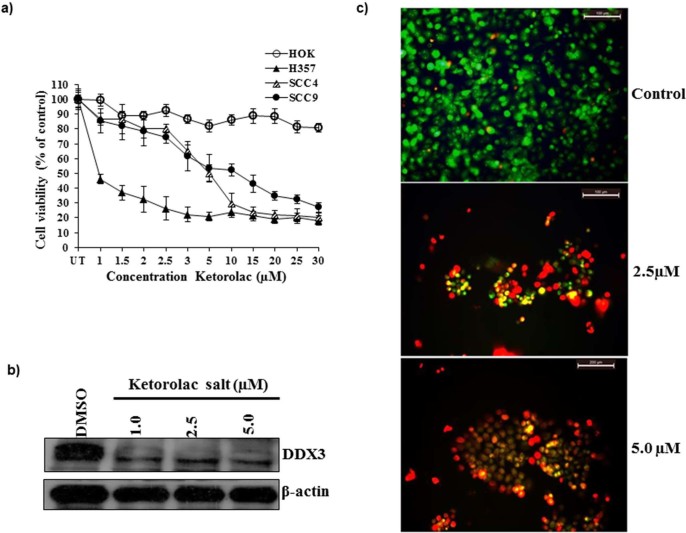
Ketorolac salt inhibits the DDX3 protein expression and reduces the cell viability in OSCC. a) HOK, SCC4, SCC9 and H357 cells were treated with indicated amount of Ketorolac salt for 48hr and cell viability was determined by MTT assay. SD (n = 3). b) Western blotting was performed to detect the expression of DDX3 and β-actin using the representative protein lysate of H357. c) Induction of apoptotic effect upon incubation of H357 cells with ketorolac salt, Green, orange and red colour indicates live, early and late apoptosis cells respectively. Cells were stained with acridine orange and ethidium bromide.

To study the anti-cancer activity of Ketorolac salt on OSCC cells, Ketorolac salt was treated to different cell lines in a dose dependent manner for 48hr and cell viability was determined by MTT assay. As shown in Figure 3a, Ketorolac salt was able to decline the cell growth from 1 µM and it continues until 30 µM concentrations. The half maximal inhibitory concentration (IC_50_) of Ketorolac salt in H357 cells is 2.6 µM, whereas the IC50 in case of SCC-4 and SCC-9 found to be v 7.1 and 8.1 µM respectively. However, the normal HOK cell line did not show any cell death effect following incubation with ketorolac salt. Further, our immunoblot study suggests that Ketorolac salt significantly reduced DDX3 protein expression levels, but not completely ablated as compared to DMSO treated cells (Figure 3b). Based on the IC_50_ values the H357 cells were treated with 2.5 and 5.0 μM concentration of Ketorolac salt and cells were stained with acridine orange and ethidium bromide. As shown in Figure 3c, bright red fluorescent cells were observed in drug treated cells as compared to vehicle treated cells indicating cell death. Collectively, these results suggest that Ketorolac salt effectively kills the cancer cells and inhibits the expression of DDX3 protein.

### Ketorolac salt Inhibit the proliferation of Oral cancer cells

**Figure 4:**
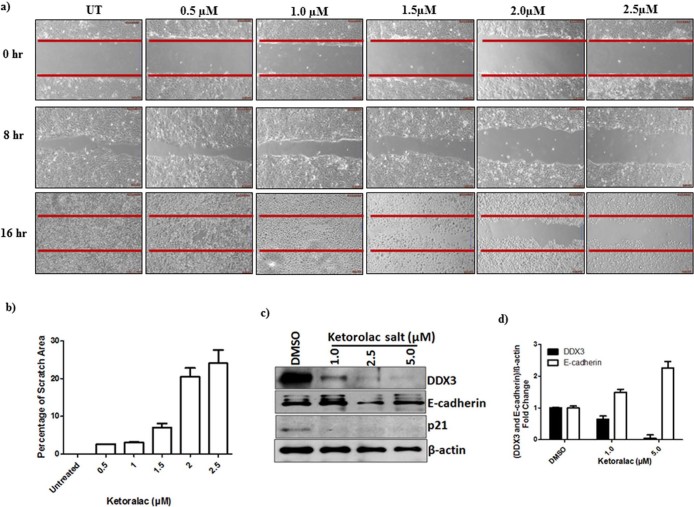
Ketorolac salt inhibits the growth of human OSCC: a) H357 cells were placed on 6 well culture plates. After the cells became confluent (monolayer) scratch was made in each well with the help of a 10 μL micropipette tip to generate a uniform wound that was devoid of adherent cells. Wells were treated with DMSO and with indicated concentration of Ketorolac salt. The photograph was taken using inverted microscope b) Bar graph showing quantification of the scratch area. c) H357 cells were treated with indicated amount of Ketorolac salt for 48h and lysate was prepared, western blotting was performed to detect the expression of DDX3, E-cadherin, p21 and β-actin. d) The Bar graph indicates the ratio of DDX3 and E-cadherin to β-actin band intensity. SD (n = 3).

To test whether the Ketorolac salt could decrease the migration of H357 cells, we employed a scratch/wound assay. The percentage inhibition of cell proliferation is calculated based on the cleared area at 16 hr using the TScratch Software (http://www.cse-lab.ethz.ch). Scratch analysis showed that the percentage of cleared area in untreated cell is 0%, whereas, the percentage of cleared area in Ketorolac salt treated cultures is continuing to increase up to 2.5 µM concentration and it showed 24% of the cleared area (Figure 4a&b). These results, therefore, indicate that Ketorolac salt can effectively inhibit the proliferation of oral cancer cells.

### Functional validation of DDX3-Ketorolac salt complex

To address whether inhibition of cancer cell proliferation and growth by Ketorolac salt is DDX3 specific, the expression of DDX3 was scored for its downstream targets such as cell adhesion protein, E-cadherin and p21 by measuring their protein levels^11^. As demonstrated in Figure 4c Ketorolac salt decreased the expression levels of DDX3 and up regulated the expression of E-cadherin at 1.0 µM concentration. No detectable changes are detected with respect to p21 protein, another downstream regulator of DDX3. Next we quantified the differential expression of E-cadherin protein in the presence and absence of DDX3 using ImageJ software. Ketorolac salt treated H357 cells displayed 1.8 fold up regulation of E-cadherin expression at 5 µM concentrations as compared to mock treated cells (Figure 4d). It suggests that DDX3 repressed E-cadherin expression and its down regulation by Ketorolac salt restores the expression of E-cadherin.

### Ketorolac salt directly interacts with DDX3 and inhibits the ATPase activity

**Figure 5:**
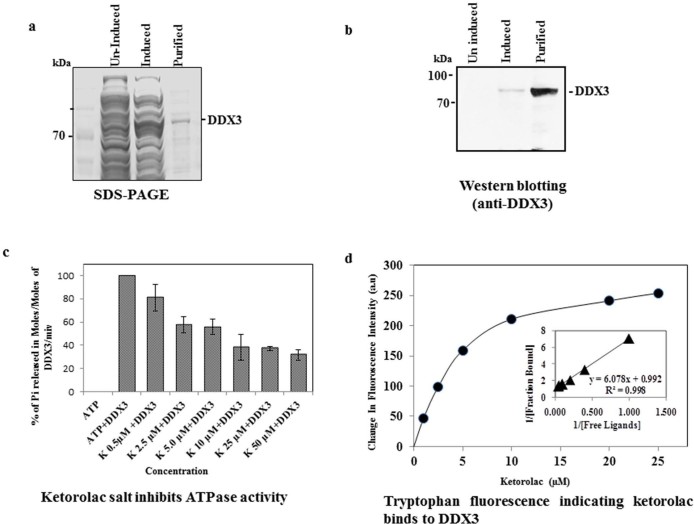
Binding assay for His-DDX3 to Ketorolac salt. a) SDS-PAGE and coomassie staining showing uninduced, induced and purified His-DDX3. b) Western blot was performed using polyclonal anti-DDX3 antibody. c) Binding of Ketorolac salt to His-DDX3 results in decreased ATPase activity. 6 µM of His-DDX3 was incubated in the absence and with increasing concentration (0.5 µM to 50 µM) of Ketorolac salt. The Moles of Pi released per Moles of His-DDX3 per minute was determined by standard Malachite Green Amoniummolybdate assay as described in Materials and Methods. d) Binding of Ketorolac salt to His-DDX3. 1.5 µM of His-DDX3 was incubated without or with increasing concentration (1 µM, 2.5 µM,5 µM,10 µM,20 µM and 25 µM) of Ketorolac salt. The tryptophan emission intensity was measured at 345 nm and a gradual reduction in fluorescent intensity was observed. (Insert) The dissociation constant for the interaction between Ketorolac salt and His-DDX3 was obtained by the double reciprocal plot (kd: 6.0±0.5 µM).

To identify the role of Ketorolac on DDX3 ATPase activity, we cloned, expressed and purified the full length human His-DDX3 protein using a bacterial over expression system. Purity and identity of the DDX3 protein were confirmed by SDS-PAGE (SDS-polyacrylamide Gel Electrophoresis) (Figure 5a) and by immunoblot analysis using DDX3 specific antibodies (Figure 5b). Then we incubated the purified His-DDX3 (6 µM) with increasing concentrations (0.5, 2.5, 5.0, 10, 25 and 50 µM) of Ketorolac Salt and measured the ATPase activity by malachite green assay. The result indicated that the addition of Ketorolac salt to DDX3 resulted in continual decline in the inorganic phosphate (Pi) release with respect to control untreated group (Figure 5c). However, when the DDX3 protein was incubated with 2.5 µM concentration, the release of Pi was reduced by approximately 50%. This result suggests that Ketorolac salt inhibits the DDX3 ATPase activity in a dose dependent manner. Next we assessed the drug-protein interactions based on tryptophan fluorescence of DDX3. The result showed that Ketorolac salt reduced the tryptophan fluorescence of DDX3 in a concentration dependent manner (Figure 5d). The change in fluorescence intensity was used to calculate the dissociation constant of DDX3-Ketorolac salt complexes. A dissociation constant of 6.0 ± 0.5 µM was calculated using double reciprocal plot of the binding data. Overall, the above data suggests that Ketorolac salt directly interacts with DDX3 and inhibit its ATPase enzyme activity.

### Ketorolac salt reduces oral carcinogenesis

**Figure 6:**
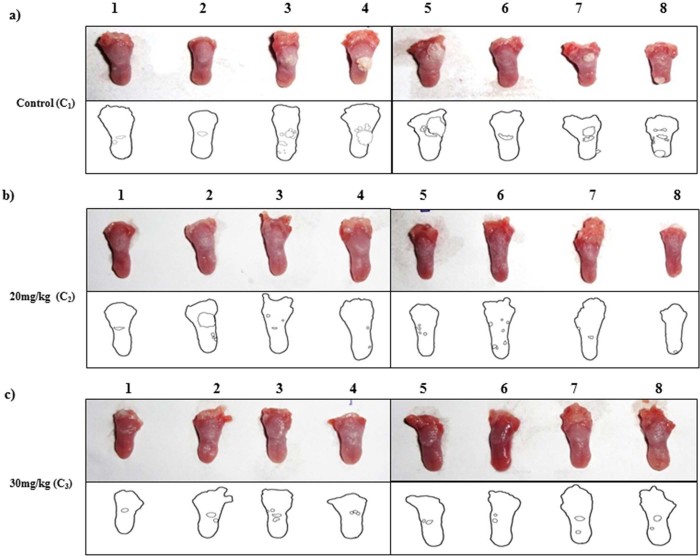
Ketorolac salt inhibits tumor growth in 4-NQO induced tongue OSCC mouse model. BALB/c mouse were administered 4-NQO orally for 20 weeks followed by mice were reverted back to normal water for next 4 weeks. After having visible tumors on the tongue, mice were randomly divided into 3 experimental groups with 8 mice in each group, i.e. of Control treated (C_1_1-8), Ketorolac treated at 20 mg/kg (C_2_1-8) and Ketorolac treated at 30 mg/kg (C_3_1-8). The drugs were injected IP twice a week for 3 weeks. At the end of the experiments mice were euthanized and tongues were photographed. Digital image of tongue and outline were represented in upper and lower panel respectively. Lower insert: Digital outlines of representative tongues of mock and Ketorolac-treated mice, showing a decreased tumor burden in the Ketorolac-treated group. **Lane a)** C_1_1-8 represent control treated (1x PBS injected) group **Lane b)** C_2_ **1-8** 20 mg/kg of Ketorolac injected group and **Lane c)** 30 mg/kg of Ketorolac injected group respectively.

**Figure 7:**
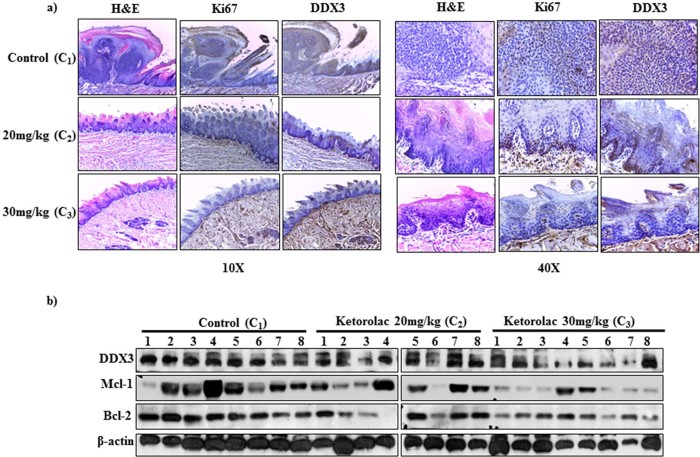
Evaluating therapeutic impact of Ketorolac salt against DDX3 induced oral cancer by Immunohistochemistry and Immunoblot analysis. Eight animals per group were used for this experiment. Following autopsy, the tongues were removed, fixed and embedded in paraffin. a) Photomicrograph of representative sections H&E staining and immunostained with DDX3 obtained at10X and 40X b) Tongue-tumor lysate proteins (20 μg/lane) were run in 10% SDS-polyacrylamide gel, electrophoresed and subsequently transferred to PVDF membrane. Immunoblots were probed with antibody for DDX3, Bcl-2, Mcl-1 and β- actin (loading control). C_1_1-8 represent the control treated C_2_ and C_3_ 1-8 represent 20 mg/kg abd 30 mg/kg Ketorolac treated groups respectively.

To biologically characterize the role of DDX3 in oral cancer biogenesis, we provided the 4-NQO orally to BALB/c mice for 20 weeks after which mice were reverted back to normal water for next 4 weeks^24^. After having visible tumors on the tongue, the animals were given IP injection with Ketorolac salt (20 mg/kg and 30 mg/kg) two times in a week for 3 weeks. At the end of the experiment, we found decreased tumor burden in case of the treated groups as compared to the control groups (Figure 6 a-c Top panel). Tumor load was outlined as a digital image (Figure 6 lower panel). To monitor the toxicity related issues, we measured the body weight; the weight remained unaffected and is unaffected throughout the course of treatment (Supplementary Figure 1). Immunohistochemistry analysis showed a strong cytoplasmic DDX3 expression in untreated control group as compared to drug treated samples (Figure 7a). Similarly, our immunoblot analysis suggests that in Ketorolac treated tumors there is reduced expression of DDX3 along with decreased expression of anti-apoptotic proteins like Bcl-2 and Mcl-1 (Figure 7b) as compared to control treated tumors.

## Discussion

DEAD box RNA helicases are capable of unwinding duplex RNA and DNA structures by disrupting the hydrogen bonds that maintain the two strands together^25^,^26^. The enzymatic activity of DDX3 has shown to be involved in replication of HCV, HBV and other family members^27^. Ring expanded nucleoside (REN), analogues have shown to inhibit the viral ATPase/ helicase activity by incorporating into nucleic acids during transcription^28^,^29^,^30^,^31^,^32^. So far, three compounds were identified as DDX3 inhibitors namely, FE-15, NZ-51 and RK33^29^,^33^,^34^. All these drugs have shown to reduce the cancer progression by down regulating the expression of DDX3^35^. However, it is well known that cytotoxic drugs possess side effects by influencing the function of normal cells. Because of the cross reactivity and side effects caused by well-known anticancer drugs, the present focus was more towards replacing these compounds by natural bioactive compounds^36^. Therefore, we have chosen Ketorolac salt from ZINC natural database based on molecular docking approach, to validate the biological activity against DDX3.

Ketorolac salt is a pyrrolizine carboxylic acid derivative and is structurally related to indomethacin^37^. The generic trademark of Ketorolac salt is *Toradol* and it belongs to the family of non-steroidal anti-inflammatory drugs (NSAIDs), which are mainly used for the treatment of inflammation and pain after surgery. Inflammation has shown to associate with numerous environmental and genetic factors^38^. Hypoxia is one of the major factors associated with inflammation in the progression of cancer^39^. Cells within hypoxia and low pH regions of solid tumors acquire aggressive chemo- and radiotherapy resistant phenotype^40^,^41^. Along these lines, DDX3 has been shown to be associated with Hypoxia and an anti-apoptotic complex within the context of TRAIL-R2 resistant MDA-MB-231, which is well established as an aggressive breast cancer cell line^19^,^42^. From the above literature, it is clear that DDX3 is one of the inflammatory markers and might be associated in tumor resistance and relapse. Ketorolac salt has shown to suppress early breast cancer relapse in relevance to triple negative subgroup^43^. It is also being used in Breast cancer surgery to improve postoperative oncological outcome (http://clinicaltrials.gov/show/NCT01806259). To identify the possible action of Ketorolac salt on DDX3, we have treated DDX3 expressing oral cancer cell line, H357 with increasing concentration of Ketorolac salt. In our experimental analysis, Ketorolac salt effectively induced cancer cell death in a dose dependent manner and down regulated the expression of DDX3. Next, we asked whether Ketorolac salt is directly interacting with DDX3 and thereby the complex is functionally active in cancer cells. For that we have biochemically validated the ATPase activity of DDX3 and excitation and emission spectra of tryptophan amino acids in presence and absence of the compound. Results showed that ketorolac salt significantly inhibited the ATP hydrolysis. Moreover, shift in the tryptophan fluorescence was also observed in a dose dependent manner. It suggests that the Ketorolac directly interact within the P-loop region (Gly 227, Gly 229 and Thr 231) of DDX3. To address the direct function we scored the expression of DDX3 downstream target proteins such as, E-cadherin and p21. Ketorolac salt induced the expression of E-cadherin in a dose dependent manner and indicates DDX3-Ketorolac salt complex is biologically more stable. To confirm the ability of Ketorolac salt in decreasing the invasion, a standard scratch assay was performed. Results showed that in the presence of Ketorolac salt, cancer cells were unable to fill the scratch area by the end of 16 hours. To validate the anti-proliferative action of Ketorolac salt *in vivo*, BALB/c mice were injected intraperitonially with Ketorolac salt and histopathological (H&E) analysis was performed. Histopathological analysis revealed that the control group displayed invasive tumor front with moderate to severe dysplasia. Dysplastic features like loss of polarity, nuclear pleomorphism, hyperchromasia, increased or abnormal mitosis were evident in the epithelial cells of H&E stained sections (Figure 7a). The degree of abnormal mitosis and the above mentioned characters were not found in case of the both the treated groups. It further supports the functional activity of DDX3-Ketorolac salt complex. Similarly, PAS staining of tongue tumor tissue section shows marked areas representing the migratory action of epithelial cells confirming the break in basement membrane along with invasion (Supplementary figure 2).Taken together, the present work demonstrated the ability of Ketorolac salt as a novel drug candidate against DDX3 associated oral cancer and other DDX3 associated disorders.

## Material and Methods

All experiments were performed in accordance with relevant guidelines and regulations.

### Receptor and ligand preparation

The three dimensional protein structure of the human DDX3 receptor (PDB ID 2I4I) with resolution of 2.20 Å complexed with AMP ligand and a total of 1, 22,163 molecules were retrieved a Protein Data Bank (PDB) and the ZINC natural database (https://zinc.docking.org/browse/catalogs/natural-products) respectively. Protein preparation wizard implemented in Maestro (Maestro, v. S., LLC; New York, NY, USA) was used to prepare the protein. Ligands were filtered to remove undesirable non-lead like compounds using the program FILTER 2.0 in Open Eye Software. The outcome compounds were passed through Pan-assay interference compounds (PAINS) filter (http://cbligand.org/PAINS/) to identify a number of substructural features to minimize the screening time for the active compounds. Finally filtered compounds were imported into Lig Prep tool, which is used for preparing ligands by optimizing geometries through OPLS-2001 Force Field^44^. All possible confirmations of ligand were generated at physiological PH ± 4 to ± 7. The generated conformers range from 1 to 100 depends upon the type of ligand. Glide XP docking output post-viewer file was used to calculate the binding free energy values of the receptor and the top 10 molecules by Prime/MM-GBSA module in the Schrödinger suite.

### Ligand docking

For ligand docking, GLIDE module version 6.1 in the Schrödinger suite, a grid-based ligand docking method with energetics, was used to generate a grid box at the centroid of the ligand. The prepared natural compounds were docked using HTVS mode to screen the DDX3 inhibitors and then top molecules were subjected to extra precision (XP) docking, a powerful docking precision present in glide docking protocol^45^. The best docked confirmation obtained from the glide XP docking were taken for molecular interaction studies exhaustively. By using the LigPlot option in maestro tool bar interactions between the protein and ligands were analyzed. The receptor ligand complex interactions were calculated based on their energy and quality of geometric contacts. The docked binding structures were ranked based on the G-scores from highest to lowest binding energy and the highly ranked were selected for further studies.

### Cell lines and culture conditions

Human OSCC lines SCC-4, SCC-9, H357, BICR 22 was obtained from European Collection of Cell culture (ECACC). FaDu, a pharynx squamous cell carcinoma cell was obtained from ATCC. Primary Human Oral Keratinocytes (HOK) was isolated from healthy gingival obtained from healthy volunteers from third molar impact surgery or biopsy as described^46^ and ethics approval for the use of human subjects was obtained from Institutional ethics committee. SCC-4 and SCC-9 cells were maintained with DMEM/F12 (Gibco.1133005) supplemented with 10% fetal bovine serum, 2 mM Glutamine and 0.4 µg/ml hydrocortisone. H357 cells were maintained with DMEM/F12 (Gibco.1133005) supplemented with 10% fetal bovine serum, 2 mM L-glutamine, 0.5 µg/ml sodium hydrocortisone succinate. FaDu cells were maintained with MEM (Gibco. 11095098) supplemented with 10% fetal bovine serum, 2 mM L-glutamine, 1 mM sodium pyruvate. BICR 22 cells were grown in DMEM (Gibco. 11995065) with 2% fetal bovine serum supplemented with 2 mM glutamine and 0.4 μg/ml hydrocortisone. HOK cells were grown in serum-free Media supplemented with 2% FBS, bovine pituitary extract 60 mg/ml and epidermal growth factor (1 ng/mL) (Life Technologies).

### Acridine orange (AO) /Ethidium bromide (EB) staining

H357 cells were seeded in 6 well plates and cultured as mentioned above. The cells were washed with Phosphate buffer saline (PBS) followed by addition of 100 μl of AO and EB (100 μg/ml, Sigma) and incubated for 15min in CO_2_ incubator. Following incubation the medium was aspirated, washed thrice with PBS. The intensity of fluorescent staining was observed and the images were captured with the help of fluorescent microscope (Leica DM IL LED inverted fluorescence microscope) using appropriate color filters.

### RT-PCR

For RT-PCR total RNA was isolated using illustraRNAspin Mini Kit (GE healthcare Lifescience.25050072). RNA was quantified and 200 ng of RNA was taken for cDNA preparation using Reverse Transcriptase Core Kit (Eurogentec.RT-RTCK-03). Primers against DDX3 and GAPDH were custom designed and the RT-PCR was done to know the expression level of DDX3, where GAPDH is used as a loading control. The primer sequences used for DDX3 (forward: 5’GGAGGAAGTACAGCCAGCAAAG3’ and reverse: 5’ CTGCCAATGCCATCGTAATCACTC 3’) and for GAPDH (forward 5' CATCACCATCTTCCAGGAGC 3' and reverse 5’ GCAGGGATGATGTTCTGGAG 3’).

### Western analysis

Cells were seeded on 6-well plate (BD Biosciences.353046) to 50% confluence after which cells were treated with indicated concentrations of Ketorolac salt. After 48 hr of incubation at 37^0^C with 5% CO_2_ cells were scrapped and protein lysates were prepared to perform immunoblotting as described^47^. The primary antibody used in this study is anti-DDX3 (Cell signaling. 8192S), E-Cadherin (Cell signaling. 3195S), p21 (Cell signaling. 2947S) and β-actin (Sigma.A2066). Immunoreactive bands corresponding to the correct molecular mass of target protein were quantified by using NIH ImageJ software. Values were normalized to internal standard GAPDH for RT-PCR data and β-actin for western blot.

### MTT assay

Cells were trypsinized with 0.25% trypsin-0.1% EDTA solution and the cells were counted using TC-10 automated cell counter (BioRad) and 1500 cells were plated in 96 well plates (BD Biosciences. 353072) for overnight. All cells were treated with indicated concentration of Ketorolac salt for 48hr and MTT was performed as described^47^. The IC50 values were determined using Microsoft Excel 2010.

### Scratch assay

H357 cells were placed on 6 well culture plates. After the cells became confluent (monolayer) scratch was made in each well with the help of a 10 μL micropipette tip to generate a uniform wound that was devoid of adherent cells. Wells were treated with control and with different concentration of Ketorolac salt. The photograph was taken using Leica DM IL LED inverted microscope.

### DDX3 protein purification

Human DDX3 ORF clone (gift from Prof. Yan-Hwa Wu Lee, Department of Biological Science and Technology, National Chiao-Tung University, Hsinchu, Taiwan) was transferred into to B*am*H1 and X*ho*I site of pET-28 a (+) expression vector and the recombinant clones was confirmed by DNA sequencing using T7 forward and reverse primers. For the purification, the DDX3 expressing bacterial pellet was resuspended in lysis buffer and lysed using French cell press (Pressure Cell Homogenizer, Stansted, Model:SPCH-10). The lysed bacterial cells were centrifuged at 20,000 rpm for 20min at 4° C after which the supernatant containing the His-tagged DDX3 was purified by affinity chromatographic method using Ni-NTA agarose resin (Invitrogen). The purity and presence of the specific protein was checked by coomassie blue staining and western blotting with anti-DDX3 antibody.

### ATPase activity assay

Malachite green assay was performed to measure the production of inorganic phosphate during ATP hydrolysis by DDX3 as described in Beuria et al^48^,^49^. 6 µM of DDX3 protein in buffer (50 mMTris-Hcl: pH 7.4) was incubated with different concentrations of Ketorolac salt for 30 min at room temperature after which 1 mM of ATP, 5 mM of MgCl_2_ was added and incubated at 37^°^C for an additional 15minutes. The hydrolysis reaction was quenched by adding 10% perchloric acid (HClO_4_) and quenched reaction mixture was centrifuged to remove aggregated proteins. 5 μl of the quenched hydrolysis reaction supernatant were incubated with 995 μl of filtered malachite green solution (0.045% malachitegreen, 4.2% ammonium molybdate, and 0.02% Triton X-100) at room temperature for 30 min, and the phosphate ions produced were determined by measuring the absorbance of samples at 650 nm (UV 1102, Techcomp). The reaction was normalized including control in absence of DDX3. Standard curve was prepared with Sodium phosphate for the quantification of inorganic phosphate.

### *In-Vivo* preclinical model against oral cancer

Institutional animal ethics committee (IAEC) approval was obtained for animal experiments. Six week old BALB/c female mice with weight range of 20–25 g were obtained from our institutional animal facility and were used to evaluate the anti-tumor efficacy of Ketorolac salt against oral cancer. A stock solution of 5 mg/ml 4NQO (Sigma-Aldrich) was prepared by dissolving in propylene glycol (Sigma-Aldrich). All the mice were given 4NQO in drinking water to a final concentration of 50 µg/ml and the water was changed twice in a week for 20 weeks after which the cages were reverted back to normal drinking water up to 4 weeks. After the appearance of visible precancerous lesions on the tongue all the mice were randomly divided into 3 experimental groups with 8 mice in each group. The experimental groups are I) Control (PBS treated) II) ketorolac treated at 20 mg/kg and III) ketorolac treated at 30 mg/kg. The Ketorolac treatment was given as IP injection twice in a week for 3 weeks. During each treatment mice were monitored for weight loss or any kind of sickness.

### Histopathological analysis

At the end of the experiment, mice were sacrificed and the tongue was immediately dissected, washed thrice with phosphate buffered saline and photographed. One portion of each tumor tissue was kept in 1X RIPA and homogenized for isolation of protein lysate and the other portion of the tumor tissue was preserved in formalin solution. Multiple sections of thickness 5 µm were cut from the tumor tissues that were processed for immunohistochemistry and H&E as described earlier^47^. All the sections were incubated with either DDX3 antibody (Abcam: ab151965) or Ki67 antibody (Vector laboratories: VP-RM04) at a dilution of 1:300. Images of slides were taken using Leica DM500 microscope at a magnification of 10X and 40X.

## Author Contributions

**Authors contributions** M.B. and R.D. designed, and drafted the manuscript. M.B., G.K.V., S.R. and S.K. S. performed experiments. All authors reviewed the manuscript.

## Supplementary Material

Supplementary Information supplementary file
