# Supplementary material for: Ketorolac salt is a newly discovered DDX3 inhibitor to treat oral cancer
Source: Sci Rep. 2015 Apr 28;5:9982. doi: 10.1038/srep09982 (PMC4412077; doi:10.1038/srep09982)
Supplement: Supplementary Information — supplementary file [file srep09982-s1.doc]

**Supplementary data**

**Ketorolac Salt Is Newly Discovered DDX3 Inhibitor to Treat Oral Cancer**

Sabindra K. Samal^1,2^, [Samapika Routray](http://www.sciencedirect.com/science/article/pii/S1368837514002267)^3^, Ganesh Kumar Veeramachaneni^4^, Rupesh Dash^1*^and Mahendran Botlagunta^4,5*^

**Material and Methods**

**In-Vivo preclinical animal model against oral cancer**

Six week old BALB/c mice with weight range of 20-25g were obtained from our institutional animal facility and were used to examine the action of Ketorolac salt against oral cancer. To induce tumors we purchased a carcinogen 4-Nitroquinoline-1-oxide (4NQO) from Sigma-Aldrich. A stock solution (5mg/ml) was prepared by dissolving in propylene glycol (Sigma-Aldrich) and was stored at 4ºC. All the mice were given 4NQO in drinking water to a final concentration of 50µg/ml and the water was changed twice in a week for 20 weeks after which the cages were reverted back to normal drinking water (without 4NQO) up to 4 weeks. After the appearance of visible precancerous lesions on the tongue all the mice were randomly divided into 3 experimental groups with 8 mice in each group. The experimental groups are I) Control (PBS treated) II) ketorolac treated at 20 mg/kg and III) ketorolac treated at 30 mg/kg. The Ketorolac treatment was given as IP injection twice in a week for 3 weeks. During each treatment mice dynamics of body weight gain or loss any kind of sickness was monitored until 21 days.

**Western blot analysis**

H357 cells were placed on 6-well plate (BD Biosciences.353046) to 50% confluence after which cells were treated with indicated molar concentrations of Ketorolac salt. Following time intervals, isolated total proteins were separated on SDS PAGE was and the immune-reactivity of DDX3, β-Actin, E-cadherin and p21 was validated in drug treated samples. Throughout the experiments the gels were run under the same experimental conditions and the full length immunoblots are depicted here.

**ADME Screening**

QikProp a module version 3.8 in Schrödinger suite was used to calculate ADME properties of the ligands. This module used to analyze the properties like human oral absorption, CNS activity, log BB, octanol/water, Caco-2, MDCK cell permeabilities, Lipinski Rule-of-Five, etc. known as pharmaceutical relevant properties which helps in making the selection of suitable ligand. Before using QikProp all the ligands need to be prepared by Lig Prep. Human oral absorption, CNS, Lipinski’s rule of 5 and log BB parameters were taken as of main interest. Human oral absorption is measured on a scale of CNS activity on a scale of -2 (inactive) to 2 (active) ,blood brain barrier crossing ability on a scale of -3.0 (unable to cross BBB) to 1.2 (capable of crossing BBB), Lipinski’s rule of 5.

**Nuclear magnetic resonance (NMR) spectroscopy:**

NMR measurements were done at (Laxai Avanti Life Sciences, Hyderabad). 1D, ^1^H and ^13^C-NMR spectra were recorded at 300º K on Avance III Bruker 400 MHz NMR spectrometer. All 1D spectra were obtained using the standard Bruker software. The samples were dissolved in deuterated solvents (DMSO-d6) and tetramethylsilane (TMS) as an internal standard; the choice of the solvent depends mostly on the solubility of the compound. Residual solvent signals of (DMSO-d6 at 2.49 ppm and 39.5 ppm) were considered as internal reference signal for calibration. The observed chemical shift values (*δ*) were given in ppm and the coupling constant (*J*) in Hz. The ^1^H and ^13^C NMR spectra were recorded under the following conditions: ^1^H NMR: acquisition time, 3.9846387 s; pulse width, 13.54 *μ*s; pulse delay, 2 s; number of scans, 24, sweep width: 8223.685 Hz, and ^13^C NMR: acquisition time, 1.3631988 s; pulse width, 9.85 *μ*s; pulse delay, 2 s; number of scans, 32000, sweep width: 24038.461 Hz, respectively. Analysis was achieved by positive ion mode using a mass spectrometer (61200 single quadrupole) in ESI mode. Some important parameters of mass spectrometer are nebulizer gas flow 60 psi, dry gas flow 5 L/min, and dry gas temp. 275°C, vaporization temp. 200°C, charging voltage 2000 V, capillary voltage 2000 V, carona 1 *µ*A. Nitrogen was used as nebulizing and collision gas. The data were collected and processed by Analyst chemstation software

**PAS staining:**

Sections were kept in xylene to remove paraffin and hydrated in ethanol (100% to 50%) and dipped in distilled water for 5 min. Then the sections were oxidized with periodic acid solution (Loba Chemie Pvt. Ltd. India) for 5 min and washed with 4 changes of distilled water. After that the sections were covered with Schiff’s reagent (Loba Chemie Pvt. Ltd. India) for 15 min at room temperature. The sections were then rinsed with running tap water for 10 minutes and nuclear staining was done by Hematoxylin (HIMIDIA) for 2 minutes. After that the sections were washed in running tap water and dehydrated using ethanol (70% to 100%), cleared with Xylene solution and mounted in permanent mounting medium (Vector laboratories).

**Supplementary Results**

To monitor the toxicity related issues caused by ketorolac salt, we measured the body weight of each BALB/c mice in grams during the whole treatment period and plotted in graphs. As shown in Supplementary Fig. 1 the weight remains same and is unaffected throughout the course of treatment even at 30mg/kg of body weight.


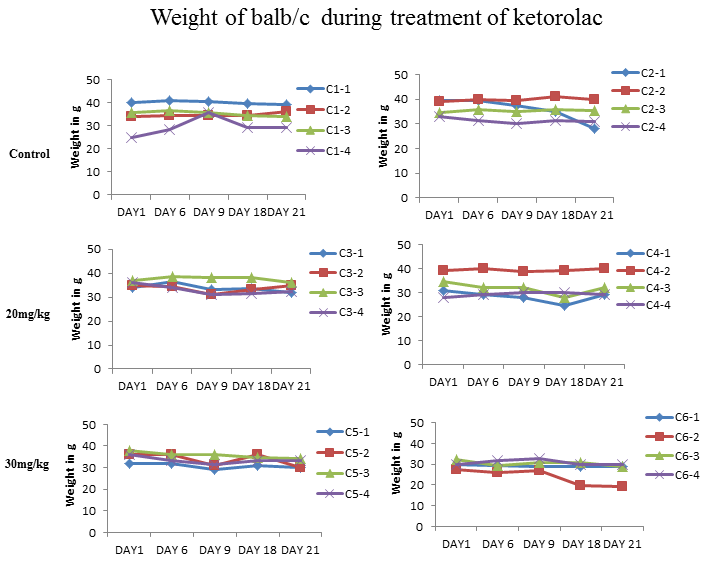


**Supplementary Fig 1:** Weight of each BALB/c mice in grams during the whole treatment period plotted in graphs. The above graphs represent the weight of BALB/c mice taken at the indicated days (Day1 to Day 21).

PAS staining


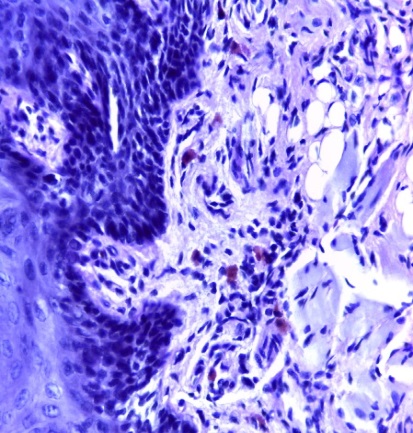


**Supplementary Fig 2:** PAS staining of control tongue tumor tissue sections. The stained section shows break in basement membrane along with invasion where marked area represents the migratory action of epithelial cells

**Original Western blots**

**Figure 2C in the main manuscript**

**
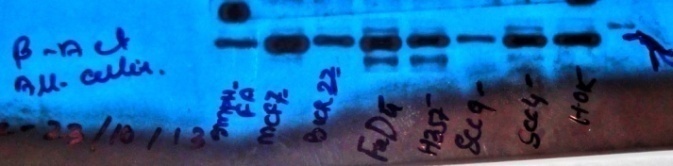
**

**Figure 3b in the main manuscript**

**Figure 4c in the main manuscript**

**Figure 5a and 5b in the main manuscript**

**
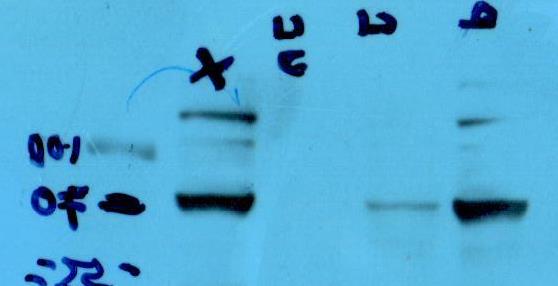

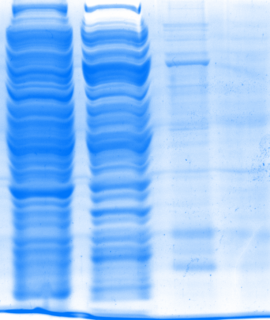
**

**Figure 7b in the main manuscript**

**ADME analysis**

All the compounds satisfy the values of partition coefficient of octanol/gas (QPlogPoct) (-2.0 to 6.5), brain/blood (QPlogBB) (-3.0 to 1.2), MDCK cell permeability (QPPMDCK) and Caco-2 cell permeability (QPPCaco-2) (<25 poor and >500 great) predicted to be blood brain and gut blood barrier, IC50 value for blockade of HERG K+ channel (QPlogHERG) (concern below – 5), drug binding to human serum albumin (QPlogKhsa) (-1.5 to 1.5) is shown in table 1.

| Molecule | CNS | QPlogPo/w | QPlogHERG | QPPCaco | QPlogBB | QPPMDCK | QPlogKhsa | % Human |
| --- | --- | --- | --- | --- | --- | --- | --- | --- |
| ZINC_ID | (-2 to +2) | (-2.0-6.5) | below -5 | <25 poor, >500 great | –3.0 to 1.2 | <25 poor, >500 great | –1.5 to 1.5 | OralAbsn >80% is high <25% is poor |
| ZINC03015344 | -2 | 2.079 | -4.014 | 24.369 | -1.817 | 17.103 | -0.367 | 63.94 |
| ZINC15673531 | -1 | 1.227 | -4.549 | 20.864 | -0.948 | 15.443 | 0.094 | 58.03 |
| ZINC04701527 | -1 | 1.943 | -1.915 | 279.148 | -0.488 | 158.416 | -0.502 | 82.1 |
| ZINC15673774 | -1 | 0.728 | -4.002 | 23.555 | -0.783 | 12.108 | 0.013 | 55.76 |
| ZINC00133268 | -2 | 2.246 | -2.789 | 58.376 | -1.343 | 29.19 | -0.29 | 71.7 |
| ZINC03881757 | -2 | 2.311 | -2.509 | 73.231 | -1.038 | 37.29 | -0.21 | 73.84 |
| ZINC00170052 | -1 | -1.028 | -2.211 | 25 | -0.422 | 12.91 | -0.6 | 45.94 |
| ZINC04042935 | -2 | 1.044 | -0.835 | 1.848 | -2.181 | 0.889 | -0.836 | 37.83 |
| ZINC05117536 | -1 | 0.989 | -3.942 | 338.07 | -0.8 | 153.2 | -0.569 | 78 |
| ZINC00336501 | 0 | 0.978 | -3.007 | 1230.41 | -0.164 | 1402.74 | -0.367 | 63.94 |
| ZINC03843403 | -2 | -0.362 | -4.613 | 50.79 | -1.817 | 19.74 | -0.65 | 55.35 |
| ZINC08579317 | 0 | 1.672 | -4.097 | 738.054 | -0.367 | 586.56 | -0.345 | 88.07 |
| ZINC20272437 | -2 | -0.238 | -2.515 | 28.76 | -1.229 | 13.58 | -0.952 | 51.66 |
| ZINC08733265 | -2 | 1.752 | -2.23 | 94.4 | -1.004 | 49.07 | -0.502 | 72.551 |
| ZINC00061866 | 2 | 4.273 | -5.429 | 6884 | 0.606 | 10000 | 0.405 | 100 |
| ZINC03847307 | 1 | 1.588 | -4.241 | 1003.28 | 0.437 | 549.246 | -0.202 | 89.96 |
| ZINC59588158 | -1 | 1.887 | -4.985 | 392.98 | -0.994 | 179.853 | -0.113 | 84.4 |
| ZINC03850500 | -2 | 0.322 | -3.519 | 4.661 | -2.22 | 1.9 | -0.496 | 40.798 |
| ZINC00011012 | -1 | 2.802 | -3.051 | 144.583 | -0.765 | 77.799 | -0.16 | 82.011 |
| ZINC03848045 | 1 | 2.854 | -3.051 | 144.583 | -0.765 | 77.799 | -0.16 | 82.011 |
| ZINC00300858 | 1 | 1.485 | -3.928 | 6248.1 | 0.333 | 3584.754 | -0.06 | 100 |
| ZINC00404428 | 0 | -0.31 | -3.876 | 2111.705 | 0.002 | 1109.789 | -0.374 | 95.148 |
| ZINC00224430 | 0 | 2.671 | -4.79 | 254.957 | -0.237 | 124.937 | -0.371 | 75.653 |
| ZINC03847253 | -1 | 2.502 | -5.048 | 1469.226 | -0.3 | 749.813 | 0.054 | 100 |
| ZINC68572775 | 1 | 1.064 | -4.937 | 798.265 | -0.776 | 387.778 | 0.015 | 93.537 |
| ZINC00519369 | 0 | 1.513 | -2.065 | 3477.887 | 0.312 | 2751.144 | -0.748 | 96.556 |
| ZINC68602672 | -1 | 0.959 | -3.517 | 1357.998 | -0.372 | 688.649 | -0.615 | 91.875 |
| ZINC00261855 | 1 | 1.656 | -3.669 | 412.543 | -0.822 | 330.902 | -0.504 | 79.375 |
| ZINC11535860 | 0 | 2.489 | -4.616 | 2354.414 | 0.029 | 1248.278 | -0.477 | 96.995 |
| ZINC00270424 | 1 | 2.831 | -3.722 | 2831.783 | 0.096 | 1523.959 | 0.196 | 100 |
| ZINC68602637 | 0 | 0.781 | -3.265 | 1545.177 | -0.193 | 791.796 | -0.725 | 88.595 |
| ZINC03845223 | -2 | 0.915 | -0.862 | 1.453 | -2.389 | 0.686 | -0.914 | 35.209 |
| ZINC01719357 | -1 | 2.547 | -4.792 | 651.497 | -0.815 | 311.323 | 0.05 | 92.225 |
| ZINC68572801 | 0 | 2.864 | -4.553 | 2922.209 | -0.083 | 1576.626 | 0.023 | 100 |
| ZINC03852577 | -1 | -0.085 | -3.305 | 224.879 | -0.853 | 98.602 | -0.682 | 68.546 |
| ZINC01460501 | 1 | 3.192 | -4.383 | 5023.519 | 0.31 | 2831.762 | 0.053 | 100 |
| ZINC00038450 | 1 | 3.52 | -5.785 | 5031.585 | 0.097 | 2836.677 | 0.261 | 100 |
| ZINC68604207 | -2 | 0.847 | -3.87 | 193.308 | -1.144 | 83.728 | -0.434 | 72.824 |
| ZINC02002143 | 1 | 1.229 | -4.345 | 663.237 | 0.415 | 351.136 | -0.228 | 84.642 |
| ZINC03848062 | 1 | 2.422 | -3.731 | 3774.562 | 0.211 | 2079.096 | -0.082 | 100 |
| ZINC03848063 | 1 | 2.422 | -3.731 | 3774.562 | 0.211 | 2079.096 | -0.082 | 100 |
| ZINC03850772 | -1 | 1.331 | -4.105 | 974.845 | -0.244 | 1199.165 | -0.441 | 88.233 |
| ZINC12340765 | -2 | 0.592 | -3.873 | 259.142 | -1.117 | 114.935 | -0.544 | 73.61 |
| ZINC04006338 | -2 | 0.666 | -2.206 | 63.669 | -1.113 | 78.172 | -0.538 | 63.131 |
| ZINC03844518 | -1 | 0.91 | -4.917 | 454.434 | -0.916 | 320.319 | -0.855 | 79.837 |
| ZINC03847852 | 1 | 3.382 | -4.997 | 3968.391 | 0.203 | 2194.731 | 0.247 | 100 |
| ZINC03847853 | 1 | 3.382 | -4.997 | 3968.391 | 0.203 | 2194.731 | 0.247 | 100 |
| ZINC00154653 | -1 | 1.127 | -3.975 | 1017.284 | -0.371 | 503.958 | -0.491 | 87.373 |
| ZINC00143896 | -1 | 1.634 | -4.088 | 253.419 | -0.923 | 275.413 | -0.478 | 79.54 |
| ZINC03844912 | 0 | 1.055 | -2.756 | 1076.252 | -0.188 | 535.606 | -0.297 | 87.391 |
| ZINC03843480 | 1 | 3.054 | -5.203 | 4090.167 | 0.223 | 2267.617 | -0.08 | 100 |
| ZINC03848610 | 0 | 3.597 | -4.549 | 2256.813 | -0.136 | 2000.902 | 0.291 | 100 |
| ZINC16982849 | 0 | 1.806 | -4.113 | 1512.471 | -0.205 | 773.696 | -0.197 | 94.43 |
| ZINC00209685 | 0 | 0.873 | -3.579 | 811.146 | -0.487 | 394.545 | -0.524 | 84.127 |
| ZINC00120515 | -1 | 0.787 | -3.844 | 716.404 | -0.494 | 344.979 | -0.529 | 82.653 |
| ZINC00039461 | -1 | 0.277 | -2.811 | 400.761 | -0.544 | 521.978 | -0.673 | 75.155 |
| ZINC00781950 | 0 | 2.094 | -3.913 | 2437.477 | -0.024 | 1295.946 | -0.101 | 100 |
| ZINC02944919 | -1 | 0.305 | -2.752 | 379.669 | -0.886 | 173.675 | -0.621 | 74.899 |
| ZINC01871561 | -2 | -1.222 | -2.502 | 1.205 | -1.549 | 0.62 | -0.765 | 21.239 |
| ZINC01871563 | -2 | -1.222 | -2.502 | 1.205 | -1.549 | 0.62 | -0.765 | 21.239 |
| ZINC03847516 | -2 | -1.222 | -2.502 | 1.205 | -1.549 | 0.62 | -0.765 | 21.239 |
| ZINC03204425 | -2 | -1.222 | -2.502 | 1.205 | -1.549 | 0.62 | -0.765 | 21.239 |
| ZINC68588802 | -1 | 2.516 | -2.727 | 131.635 | -0.615 | 281.096 | -0.54 | 79.609 |
| ZINC01576954 | -1 | -0.22 | -3.451 | 263.826 | -0.998 | 117.182 | -0.731 | 68.997 |
| ZINC00001209 | 1 | 2.308 | -3.897 | 2264.692 | 0.125 | 2629.868 | -0.228 | 100 |
| ZINC03153824 | -2 | 0.444 | -0.897 | 2.256 | -1.764 | 1.361 | -1.013 | 35.869 |
| ZINC03850680 | 1 | 1.047 | -4.704 | 379.608 | 0.078 | 192.106 | -0.333 | 79.242 |
| ZINC12341384 | 1 | 2.278 | -4.026 | 2639.019 | 0.017 | 1412.146 | -0.186 | 100 |
| ZINC03844799 | 0 | 2.426 | -3.474 | 2781.647 | -0.066 | 1494.817 | -0.062 | 100 |
| ZINC03844800 | 0 | 2.426 | -3.474 | 2781.647 | -0.066 | 1494.817 | -0.062 | 100 |
| ZINC01576937 | 0 | 1.311 | -4.569 | 763.138 | -0.615 | 369.367 | -0.4 | 86.214 |
| ZINC00245868 | -1 | 3.092 | -3.213 | 151.815 | -0.774 | 82.014 | -0.007 | 84.093 |
| ZINC00245871 | -1 | 3.092 | -3.213 | 151.815 | -0.774 | 82.014 | -0.007 | 84.093 |
| ZINC03844067 | -1 | 2.874 | -1.716 | 474.516 | -0.177 | 281.095 | -0.123 | 91.672 |
| ZINC03844069 | -1 | 2.874 | -1.716 | 474.516 | -0.177 | 281.095 | -0.123 | 91.672 |
| ZINC03844070 | -1 | 2.874 | -1.716 | 474.516 | -0.177 | 281.095 | -0.123 | 91.672 |
| ZINC03844068 | -1 | 2.874 | -1.716 | 474.516 | -0.177 | 281.095 | -0.123 | 91.672 |
| ZINC12370913 | -1 | 1.834 | -2.287 | 66.489 | -0.909 | 33.598 | -0.358 | 70.305 |
| ZINC03852636 | -1 | 0.679 | -4.038 | 305.152 | -0.785 | 137.143 | -0.436 | 75.391 |
| ZINC03847057 | 1 | 2.078 | -4.824 | 4277.946 | 0.248 | 2380.349 | -0.362 | 100 |
| ZINC02144633 | 0 | 1.082 | -4.614 | 542.14 | -0.626 | 255.244 | -0.578 | 82.214 |
| FE-15 | 0 | 4.346 | -6.148 | 1322.072 | -0.367 | 5988.942 | 0.275 | 100 |
| NZ-51 | -2 | 5.816 | -6.607 | 166.853 | -3.137 | 71.414 | 1.026 | 87.817 |
| RK33 | -2 | 2.896 | -6.189 | 601.108 | -1.127 | 285.38 | -0.256 | 93.638 |

**Supplementary Figure 3: NMR and MS analysis**

Purity of the Ketorolac salt was verified by NMR analysis. The NMR spectrum of this compound was cross checked with chemdraw software. Experimental NMR data matched with graphical peak data for the single compound, suggests the 100% compound purity. Moreover, the mass spectrum of the ketorolac salt is 256.17 and in agreement with molecular weight of the compound 255.26

A)

Carbon NMR of Ketorolac Salt


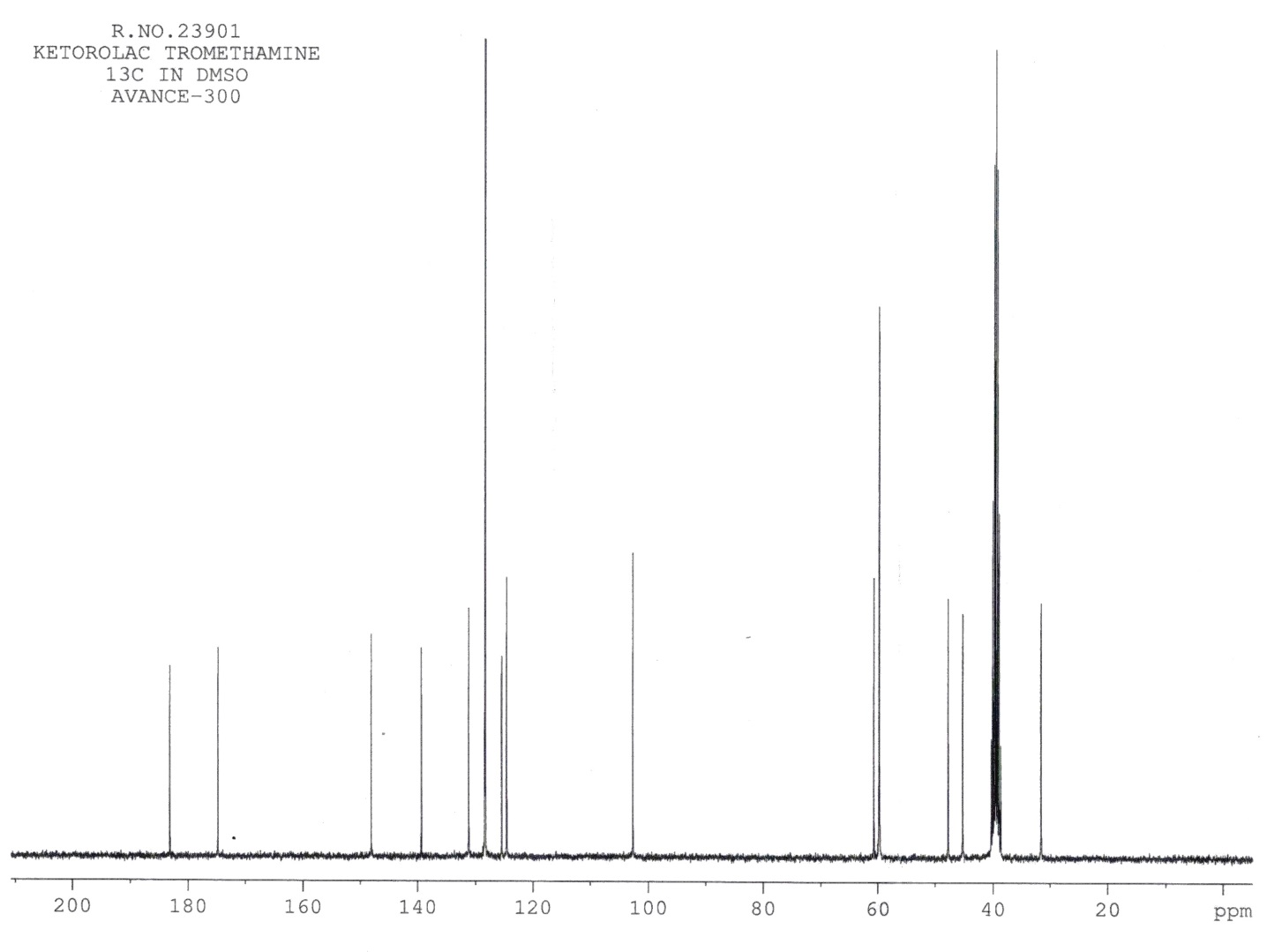


Proton NMR of Ketorolac Salt

**
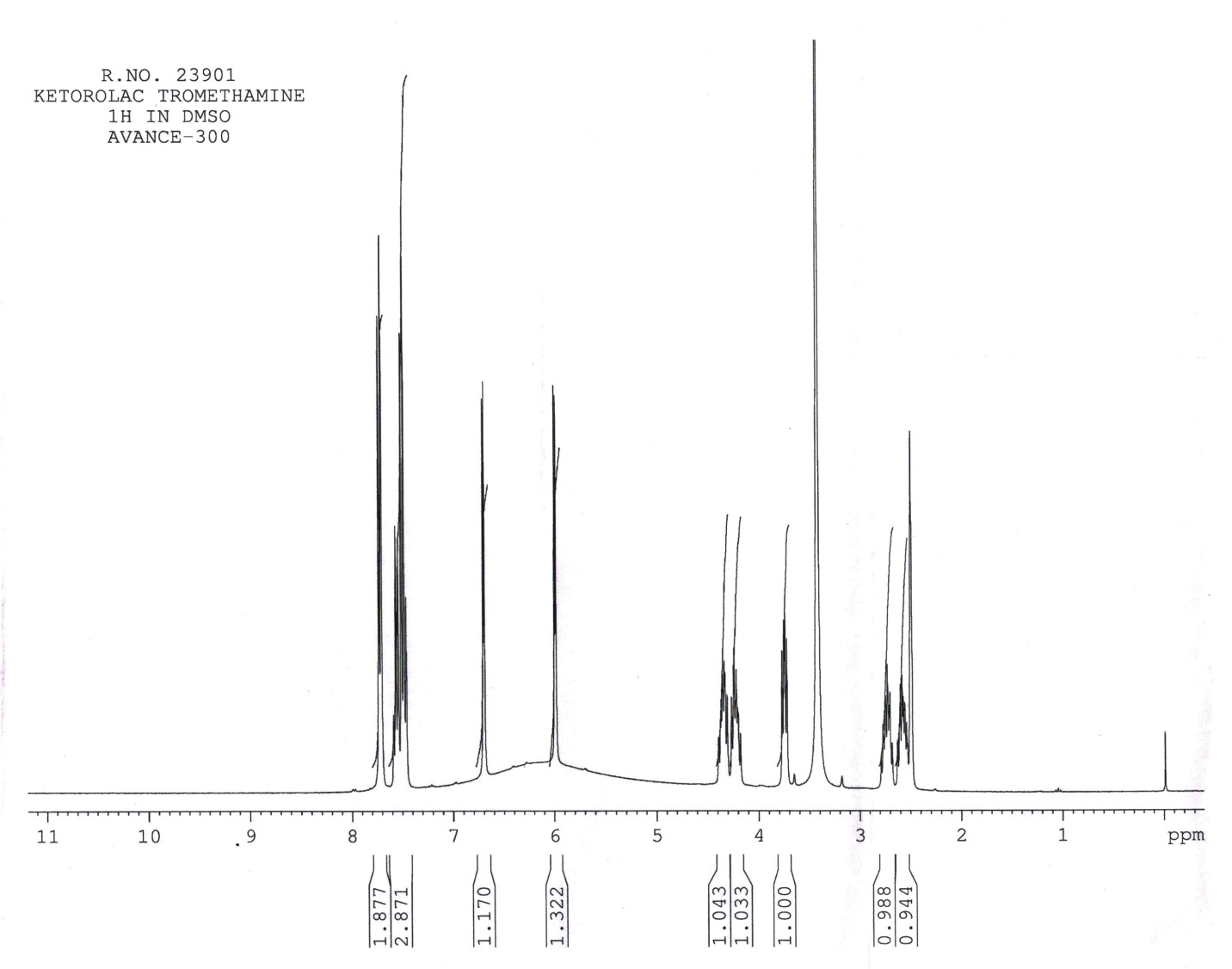
**

**B) Mass spectroscopy data**

**
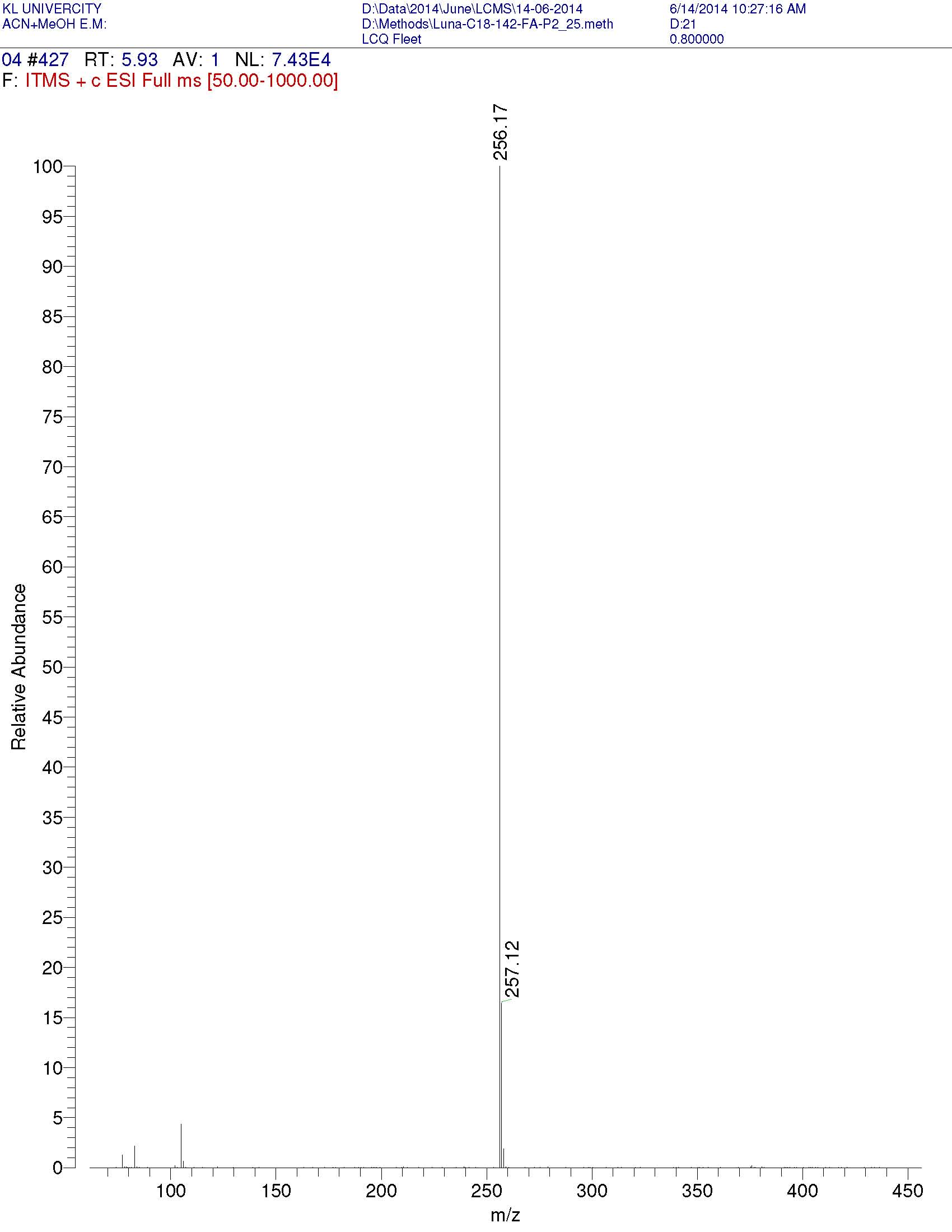
**
